# Efficacy of a Parent-Based, Indicated Prevention for Anorexia Nervosa: Randomized Controlled Trial

**DOI:** 10.2196/jmir.9464

**Published:** 2018-12-14

**Authors:** Corinna Jacobi, Kristian Hütter, Ulrike Völker, Katharina Möbius, Robert Richter, Mickey Trockel, Megan Jones Bell, James Lock, C Barr Taylor

**Affiliations:** 1 Klinische Psychologie & E-Mental Health Institut für Klinische Psychologie und Psychotherapie Universität Dresden Dresden Germany; 2 Klinik für Psychiatrie und Psychotherapie Städtisches Klinikum Görlitz Görlitz Germany; 3 Klinik für Psychosomatische Medizin und Psychotherapie Städtisches Klinikum Görlitz Görlitz Germany; 4 Sächsische Bildungsagentur Regionalstelle Leipzig Leipzig Germany; 5 Department of Psychiatry Stanford University School of Medicine Stanford, CA United States; 6 Headspace Santa Monica, CA United States

**Keywords:** anorexia nervosa, indicated prevention, internet, parental intervention, randomized controlled trial

## Abstract

**Background:**

Web-based preventive interventions can reduce risk and incidence of bulimia and binge eating disorders among young high-risk women. However, their specific effects on core symptoms of anorexia nervosa (AN) are rather weak.

**Objective:**

The primary objective of this study was to evaluate the efficacy of an indicated, parent-based, Web-based preventive program Eltern als Therapeuten (*E@T*) in reducing risk factors and symptoms of AN.

**Methods:**

Girls aged between 11 and 17 years were screened by selected risk factors and early symptoms of AN. At-risk families were then randomized to E@T or an assessment-only control condition. Assessments took place at pre- and postintervention (6 weeks later) and at 6- and 12-month follow-up (FU).

**Results:**

A total of 12,377 screening questionnaires were handed out in 86 German schools, and 3941 including consent returned. Overall, 477 (447/3941, 12.10%) girls were identified as at risk for AN and 256 of those could be contacted. In all, 66 families (66/256, 25.8% of those contacted) were randomized to the E@T or a wait-list control condition, 43 (43/66, 65%) participated in postassessments, and 27 (27/66, 41%) in 12-month FUs. Due to low participation and high dropout rates of parents, recruitment was terminated prematurely. At 12-month FU, girls’ expected body weight (EBW) percentage was significantly greater for intervention participants compared with control participants (group by time interaction beta=21.0 [CI 5.81 to 36.13], *P*=.007; group by time squared interaction beta=−15.5 [CI −26.6 to −4.49], *P*=.007; estimated Cohen *d*=0.42]. No other significant effects were found on risk factors and attitudes of disturbed eating.

**Conclusions:**

Despite a significant increase in girls’ EBW percentage, parental participation and adherence to the intervention were low. Overall, parent-based, indicated prevention for children at risk for AN does not seem very promising, although it might be useful for parents who engage in the intervention.

**Trial Registration:**

International Standard Randomized Controlled Trial Number (ISRCTN): 18614564; http://www.isrctn.com/ISRCTN18614564 (Archived by WebCite at http://www.webcitation.org/74FTV1EpF).

## Introduction

### Background

Anorexia nervosa (AN) is a serious condition with a prevalence estimated between 0.3% and 0.7% among adolescent females based on Diagnostic and Statistical Manual of Mental Disorders-Fourth Edition (DSM-IV) criteria [1-4]. AN can be accompanied by severe medical complications, including significant growth retardation, pubertal delay or interruption, and peak bone mass reduction [5,6]. Furthermore, the mortality rates associated with AN are significantly elevated when compared with standard population norms [7]. Approximately 60% of all eating disorder patients have a lifetime affective disorder [8], 35% of AN patients also suffer from obsessive-compulsive disorder, and there is a moderate overlap of AN and avoidant personality disorder. Furthermore, physical and psychological functioning and distress can be as severe in adolescents with AN regardless of presenting weight [9]. In addition, AN is associated with increased health care utilization and health care costs [10-12]. Krauth et al [12] reported yearly overall costs for AN of €195.4 million in Germany (€64.9 million through hospitalization, convalescence benefits, and rehabilitation as well as €130.5 million indirect costs through inability to work and premature death).

Given the seriousness of the disorder, the poor prognosis, and the associated burden and costs, early preventive interventions are of crucial importance. If these interventions target modifiable and potent risk factors, this could reduce both the onset of the disorder or mitigate core symptoms of the disorder before the onset. A few longitudinally assessed risk factors for eating disorders have been identified [13,14], but not all are suitable for preventive approaches, for example, pre- and perinatal risk factors have been confirmed in several studies but are not modifiable, and early childhood health and eating problems have also been confirmed in several studies but would not be suitable targets of preventive interventions for older children or young adults.

The factors weight and shape concerns and dieting, on the other hand, represent the most potent, modifiable, and confirmed risk factors for eating disorders in general. However, these factors are not specific for distinct eating disorder diagnoses, such as AN. In addition to these longitudinally assessed risk factors, a number of probable (retrospectively assessed) risk factors were found. At the start of the study, perfectionism and obsessive-compulsive symptoms seemed to be the best candidates as they both were modifiable and showed some specificity for AN [13-15].

### Prior Work

Several previous reviews and meta-analyses suggest that preventive interventions for eating disorders (ED) in general reduce risk factors for, symptoms of, and—in few cases—even onset of mostly bulimic or binge eating-type ED [16-21]. Prevention programs reviewed in these analyses include the whole range of interventions from universal to indicated programs, school-based versus individually based, and are directed at age ranges from younger children to young adults. Effect sizes of the core risk factor outcomes range from low to high depending on the selection criteria applied and included samples, data analysis, and consideration of sensitivity analyses.

Although most of these meta-analyses included technology or Web-based interventions to some degree, 2 explicitly addressed effects of technology- or Web-based interventions only [16,20] with slightly different results. Beintner et al [16] conducted a cross-cultural comparison of 10 randomized controlled trials using the Web-based prevention program *Student Bodies* and found small to medium mean post and follow-up (FU) effects on drive for thinness, negative body image, and weight concerns. Loucas et al [20] found overall small post and FU effects on drive for thinness, weight and shape concern, and dietary restraint in 8 of the 13 Web-based prevention trials, including the Student Bodies intervention, and small or inconclusive effects for interventions in the remaining studies.

However, in the absence of confirmed risk factors for specific ED diagnoses, preventive interventions in general are usually not specifically directed at individuals at risk for specific ED diagnoses, such as AN but rather ED in general. This lack of diagnostic specificity of interventions is even more evident when only targeted interventions for individuals at risk are considered or moderators for intervention types are analyzed [18]. Participants in these studies are usually selected based on nonspecific risk factors for ED such as weight concerns, dieting, or body dissatisfaction. Early symptoms of specific ED categories (eg, subthreshold binge eating, compensatory behaviors, and body mass index [BMI]) are rarely used for selection. On the basis of the studies included in 1 meta-analysis [18], the mean BMI of young adult participants in these interventions was 23.3, and selection criteria did not include low BMI to determine risk status. It therefore seems likely that individuals at higher risk for AN were not reached by these interventions, and tailored preventive interventions for these individuals need to be developed.

Current treatment approaches for adults with AN have shown only limited effects [22], but there is considerable evidence supporting the effectiveness of family-oriented treatments [23-25] for adolescents. Family-based treatment (FBT [26]) has also been recommended by the American Psychiatric Association (APA [27]) and the National Institute for Health and Care Excellence (NICE) guidelines [28] as first-line treatment for adolescents with AN. A preventive approach, targeting risk factors and early symptoms for AN combined with elements of FBT, could therefore be beneficial in preventing the onset of the disorder in high-risk adolescents.

Thus, as part of a pilot study for a subsequent randomized controlled trial, we developed a family-based intervention called *Parents Act Now* targeting individuals at risk for AN. The 6-week intervention was directed at parents, originally developed in the United States, and subsequently translated into German (*Eltern als Therapeuten or E@T*). The pilot study, conducted in parallel in the United States and Germany, examined the feasibility, acceptability, as well as short-term effects of the intervention in 46 adolescent females aged 11 to 17 years [29]. Overall, 11% of girls screened at the Germany site and 24% of girls screened at the US site met the risk criteria for AN. Parents accessed the majority of the Web-based sessions and rated the program favorably. At postassessment, we found a reduction in risk status for 16 out of the 19 participants. Participants remained stable or reported increased EBW percentage and decreased eating disorder attitudes and behaviors. However, the pilot study was also characterized by parents’ rather low willingness to participate in and low compliance with the intervention. To address these problems, we made a number of changes to the intervention itself (eg, addressing potential denial and downplaying of eating problems in the first session) and the assessment procedure (eg, adding a motivational enhancement module to the first assessment where parents received feedback on the risk status of their daughters) before conducting the main study.

### Goal of This Study

Following this pilot study, the major objective of this study was to determine the efficacy of the parent-based, Web-based, indicated preventive intervention *E@T* in comparison with a wait-list control group. We hypothesized that children of parents participating in the intervention would show an improvement in core AN symptoms, that is, weight loss, overvaluation of weight and shape, and restraint eating.

## Methods

### Design

We conducted a randomized controlled trial including parents and their daughters recruited from schools in Saxony, Germany. Eligible participants were randomized either to E@T or a wait-list control group. Assessments took place before randomization (at baseline, T1), at postintervention (6 weeks after baseline, T2), and at 6- and 12-month FU (8 [T3] and 14 [T4] months after baseline). Baseline, postintervention, and FU assessments were—with few exceptions—conducted in face-to-face settings. Both parental and child consents were required.

### Participants

To be included in the study, girls had to be aged between 11 and 17 years and fulfill criteria of being at risk for AN based on the screening results. We defined at risk as a combination of factors selected from the following 3 categories [15]: (1) A: established risk factors for AN as high weight and shape concerns and drive for thinness (defined by either scoring ≥42 on the Weight Concerns Scale [30,31] or ≥24.1 on the Eating Disorder Inventory (EDI-2) subscale Drive for Thinness [32]), (2) B: early symptoms of AN indicated by low weight (defined as <90% EBW; Centers for Disease Control and Prevention, 2001) or significant weight loss (5% in the past 6 months), and (3) C: the presence of 1 out of the 4 probable risk factors, for example, high levels of perfectionism defined by scoring ≥78.0 on the Frost Multidimensional Perfectionism Scale [33], amenorrhea, excessive exercise, and a family history of an eating disorder. To be included, criterion B was mandatory and either criterion A or C (or both) was additionally required. In a previous study [15], the overall prevalence of the combination of these factors in a sample of 1562 adolescent girls was 10.8% and it increased from 9.5% to 16.5% between ages 11 and 16 years.

Exclusion criteria were the presence of a full-syndrome eating disorder in the past 6 months, current major depression, current substance abuse or dependence, and suicidal ideation.

### Procedures

We asked the authorities of the school district of Saxony, Germany, for permission to conduct screenings in all high schools and secondary high schools throughout Saxony. Following their consent, 170 schools were individually invited to participate. Recruitment was completed in 86 schools (34 high schools and 52 secondary high schools) and followed a 2-step procedure. First, high-risk girls were identified through screens in participating schools after a short introduction of the study in class provided by trained research assistants. Questionnaires, including screening questions to be filled out by the children and few questions to be filled out by parents (daughters’ current weight, height, and weight loss in the past 6 months, family history of eating disorders, internet access, and willingness to participate in an internet prevention program), were completed at home, and consent forms and questionnaires were collected approximately 1 week later in schools. If children screened positive, parents and children were invited for face-to-face baseline assessments. During these assessments, we conducted separate interviews with parents and children to assess children’s eating and general pathology, to exclude ED diagnoses, and to obtain parental demographic information (education level, occupation, marital status, daughter’s number and age of siblings, and daughter’s type of school and current grade). Because the results of the pilot study suggested some problems with parental motivation, we included a manual-based motivational assessment and enhancement module (adapted from motivational interviewing) to guide interviewers’ feedback to this first assessment. Children also filled out a number of self-report questionnaires. Children’s height was measured to the nearest millimeter using a calibrated stadiometer and weight was measured to the nearest 0.1 kg using a digital scale.

Interview results were directly entered into a database (*MACRO*), which also contained algorithms to determine probable ED and other diagnoses and conduct randomization. Following separate interviews, 1 interviewer provided detailed verbal feedback on the daughter’s risk factor status, eating disorder, and general pathology to parents and the daughter together and discussed inconsistent results from both interviews with them. Interviews were conducted by experienced graduate students and by research assistants who had received intensive training before conducting the interviews. To improve and maintain interview quality, all interviews were recorded and interviewers received verbal feedback on the recordings by an experienced graduate student. In a second step, parents of eligible children were randomly assigned to the E@T intervention or the assessment-only control group.

The study was approved by the local human subjects’ committee (#EK172052010). Due to an oversight, the trial registration was delayed while participant recruitment had already started, following adaptations made to the intervention itself and the planned procedures. Participant recruitment and post and FU assessments took place between October 2010 and May 2014.

### Intervention Eltern als Therapeuten (E@T)

The parental intervention is based on the first phase of the family-based treatment for AN by Lock [26], the parent guide by Lock and Le Grange [34], and an internet-based intervention to prevent eating disorders for adolescents [35]. The intervention *E@T* consists of a 6-session Web-based program for parents accessible over the course of 6 weeks and moderated by eating disorder experts (graduate-level clinical psychologists in training under supervision). The intervention also includes a moderated Web-based discussion group for parents, weekly monitoring journals related to their daughter’s weight, eating and exercise with feedback provided by moderators, videos, and 2 phone calls to enable individualized feedback on the daughter’s problems with eating, weight and shape concerns, and referral to other resources if necessary. Adolescents received a brief handout describing the purpose of the study written for a general audience in clear, lay terms, at a 6-grade reading level. A more detailed description of the intervention is summarized in a previous report [29].

### Measures

#### Screening

The screening questionnaire consisted of 61 questions covering established risk factors, possible risk factors, and early symptoms of AN: weight and shape concerns based on the Weight Concerns Scale (WCS), a 5-item self-report screening questionnaire to identify students at risk for developing an ED [31]. Previous studies have shown that 10% of girls in the highest quartile of the WCS subsequently develop a subthreshold or full-syndrome ED. The German validation of the WCS [30] has a high test-retest reliability (*r*=.95).

In addition, drive for thinness was based on the respective 7-item subscale of the EDI-2 [32,36], self-reported height and weight, weight loss, and the presence of an ED in the past 6 months; perfectionism was based on the Frost Multidimensional Perfectionism Scale (MPS-F) [33,37]. The EDI-2 drive for thinness subscale has been shown to have high internal consistency (Cronbach alpha=.88). The MPS-F consists of 35 items covering 6 subscales (with 4 to 9 items each). The internal consistency for the subscales (Cronbach alpha) varies between .70 and .90 [33]. Questions related to the presence of secondary amenorrhea, excessive exercise, and family history of an ED in at least one family member were also included in the screen. Secondary amenorrhea was assessed by asking whether girls’ menses had already started and, if so, whether they had missed menses in the past 3 months and if they took contraceptive medication. Amenorrhea was coded *yes* if menses had started but had been missed in the past 3 months or if menses had started but the use of contraceptives was endorsed. To endorse excessive exercise, girls had to indicate that they exercised in the past 4 weeks to lose weight, to influence body shape or body fat, to burn more calories, and to receive an average score of 3 (*sometimes*) or lower on a 1 (*always*) to 5 (*never*) scale asking if they were afraid of becoming upset or if they were feeling guilty when they had to skip exercise and if they exercised in spite of being sick or injured. These 4 questions were based on the Eating Disorder Examination (EDE) interview [38-40]. Parental history of ED was obtained from both girls and parents based on questions from the risk factor interview by Fairburn et al [41].

The primary outcomes were weight normalization (defined by change in EBW percentage, objectively measured) and other core AN symptoms, such as daughters’ self-reported weight and shape concerns (assessed by the WCS), restraint, and frequency of driven exercise based on the EDE twelfth Edition [38-40]. The EDE is a semistructured interview that measures ED psychopathology on the 4 subscales: restraint (5 items), eating concern (5 items), weight concern (5 items), and shape concern (8 items), which can be aggregated to a total score and also generates ED diagnoses based on DSM-IV (text revision) criteria. The internal consistency (Cronbach alpha) varies between .73 and .86 for the subscales and is .93 for the total score. Secondary outcomes were EDE weight concern, EDE shape concern, EDE eating concern, EDI-2 drive for thinness, and EDI-2 body dissatisfaction. The latter EDI-2 subscale also has an internal consistency of .88.

Furthermore, the Schedule for Affective Disorders and Schizophrenia for School-Age Children [42] was used to assess present and past episodes of psychopathology based on DSM-IV Axis I criteria [43] in girls. At baseline, parents completed the Parent Motivation Inventory (PMI; [44]) to assess their motivation and confidence to address their daughter’s eating problem. The PMI is a 25-item scale with a high internal consistency of .96 (Cronbach alpha) [44].

At postintervention and 6- and 12-month FU, only the EDE interview was conducted with parents on the daughter, and with daughters themselves; in addition, daughters’ height and weight were measured and daughters filled out the WCS and EDI-2 subscales drive for thinness, bulimia, and body dissatisfaction and answered questions regarding treatment utilization. Whenever possible, subjective reasons for parents declining participation in the study after initial contact and feedback on daughters’ risk status were assessed qualitatively.

We assessed adherence to the intervention by mean number of sessions opened and overall percentage of program pages opened. These data were retrieved from the program log files.

### Randomization and Masking

The randomization algorithm, which was integrated into the database MACRO, was provided by the independent Centre for Clinical Trials (Koordinierungszentrum für Klinische Studien) at TU Dresden. Children were stratified by age and EBW percentage and randomized in a ratio of 1:1 to E@T or the waiting list control condition after parents had given informed consent. Parents and psychologists involved in the moderation of the E@T program could not be masked to intervention allocation. Assessors who conducted T1 to T4 diagnostic assessments could not be blinded to the intervention condition but were neither involved in the moderation of the intervention nor in final data analyses.

### Statistical Analyses

The initial study sample size estimate was based on a power calculation for a single end-point group comparison at 12 months. Therefore, the sample size calculation was based on a 2-tailed *t* test for independent samples. For this calculation, the estimated between-group effect size at 12-month FU was *d*=0.5 between the intervention and the control group for the primary outcomes: EBW percentage and other core AN symptoms. Using these parameters, the estimated sample size needed to achieve 80% power was 64 participants per group (or a total of 128 subjects). Considering a combined noncompliance and loss to FU rate of 30%, an estimated 91 participants per group would have been required to attain an adequate sample size for this study. We subsequently employed a mixed-effects model approach to assess all group differences over time. Mixed-effects models use all available data points when participants are lost to FU. This approach is likely to render higher statistical power than a *t* test used to assess group differences at a single time point. Therefore, the a priori power analysis conducted for this study (based on an independent samples *t* test) may have overestimated the sample size needed for this study.

All analyses were conducted as intention-to-treat (ITT) analyses including all randomized participants. Differences between the intervention and the control group on primary outcomes (EBW percentage, weight and shape concerns, EDE restraint, and driven exercise) as well as secondary outcomes were tested using mixed-effects models to account for the nested data structure of 4 observations across time within individual participants [45]. Total observation time was set at 1.0. Each measurement time point was set at its corresponding fraction of 1.0. This 0 to 1 time variable was then multiplied by itself to create a variable for time squared, which enables specification of quadratic regression models assessing intervention effects on change and on rate of change in outcome variables. A group by time interaction term was specified as to estimate the effect of the intervention. A group by time squared interaction term was also specified to estimate the effect of intervention on rate of change using a quadratic regression model. Cohen *d* was calculated by dividing the mixed-effects model derived intervention effect estimate by the pooled SD of the particular measure at baseline.

Differences on screening variables between participating and nonparticipating parents and children were analyzed using 2-tailed *t* test for continuous screening variables and chi-square test for dichotomous variables. These exploratory analyses were not corrected for multiple comparisons.

All analyses were performed using the software programs SPSS 22 (Statistical Package for the Social Sciences) and HLM7 (Hierarchical Linear and Nonlinear Modeling).

## Results

### Recruitment

**Figure 1:**
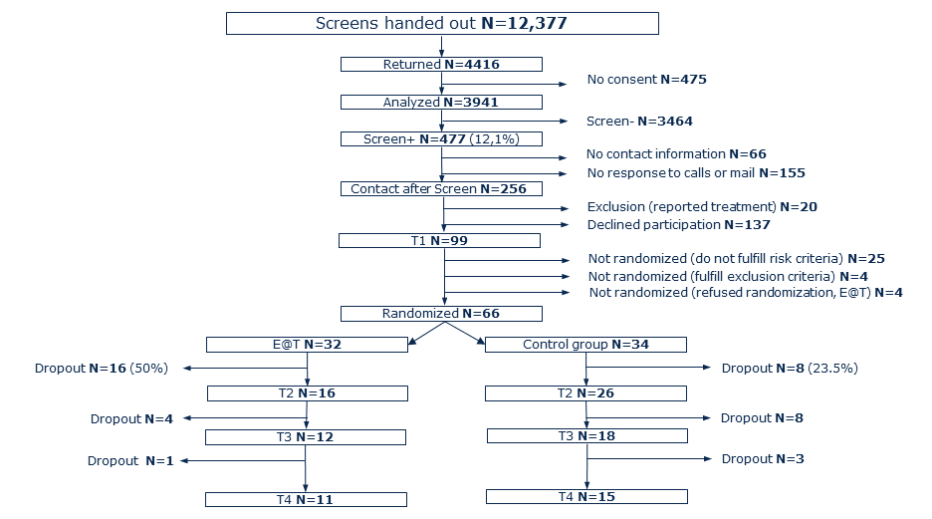
Consolidated Standards of Reporting Trials diagram of participant flow. E@T: Eltern als Therapeuten.

Between October 2010 and December 2012, a total of 12,377 screening questionnaires were handed out in schools, 4416 (4416/12,337, 35.79%) returned and 3941 (3941/12,377, 31.84%) included consent. At baseline, 99 interviews were conducted with parents and screen-positive children. In total, 33 families could not be enrolled because they did not fulfill inclusion criteria any more during these assessments (mostly because children’s EBW percentage was in the normal range when measured objectively) or refused randomization. Finally, 66 families were randomized, 32 to the E@T intervention and 34 to the control condition. At 12-month FU, the dropout rate in the E@T condition was 65.6% and in the control condition was 52.9%, and they were not significantly different. Figure 1 presents a Consolidated Standards of Reporting Trials flow diagram.

Due to a much lower response rate of parents of girls screened for risk status and the high rate of parents of at-risk girls not willing to participate, it would have taken at least twice as long to recruit the originally planned sample size. The funding of the study was therefore stopped before the originally planned sample size had been achieved, but FUs of all randomized parents were still included.

### Sample

Of participants with informed consent, 12.10% (477/3941) met predefined criteria for at risk. Overall, 47.8% of the sample fulfilled the combination of criteria A (high WCS or EDI drive for thinness), B (low weight or significant weight loss), and C (high levels of perfectionism, amenorrhea, excessive exercise, or a family history of an ED); 28.4% fulfilled the combination of criteria B and C; and 23.9% fulfilled the combination of criteria A and B. Regarding criterion B, 47% of the sample endorsed low body weight and 53% of the sample endorsed significant weight loss in the past 6 months.

Table 1 presents baseline characteristics of the sample. Included girls were on average about 14 years old, and the average EBW percentage was in the normal range. Overall, girls showed only few ED symptoms in the 4 weeks before baseline. Current or past comorbid major depressive disorder, separation anxiety disorder, social phobia, and specific phobia were also low.

**Table 1:** Sample characteristics of all randomized children at baseline.

Characteristics		E@T^a^ (N=32)	Control group (N=34)
Age in years, mean (SD)^b^		13.8 (1.5)	13.7 (1.6)
Percentage of expected body weight, mean (SD)		98.8 (12.3)	99.1 (13.4)
Objective binge episodes (past month), mean (SD)^b^		0 (0)	0 (0)
Subjective binge episodes (past month), mean (SD)^b^		0.8 (4.4)	0.1 (0.3)
Fasting (days past month), mean (SD)^b^		0.1 (0.3)	0.3 (1.2)
Vomiting (episodes past month), mean (SD)^b^		0 (0)	0.3 (1.7)
Laxative use (episodes past month), mean (SD)^b^		0 (0)	0.1 (0.5)
Excessive exercise (days past month), mean (SD)^b^		4.7 (8.6)	5.1 (9.1)
**Comorbidity, n (%)^c^**			
	Separation anxiety disorder	0 (0)	3 (9)
	History of major depression	2 (6)	2 (6)
	Social phobia	0 (0)	1 (3)
	Specific phobia	1 (3)	0 (0)

^a^E@T: Eltern als Therapeuten.

^b^According to the Eating Disorder Examination [38-40].

^c^According to Schedule for Affective Disorders and Schizophrenia for School-Age Children [42].

### Screening Differences Between Participating and Nonparticipating Parents and Children

We found no significant differences in the frequencies of endorsing screening criteria A and C between parents who participated in the study and those who refused to participate. However, children of participating parents endorsed significantly more of all 3 screening criteria (52.1% vs 37.2%; *P*=.02) and showed significantly higher levels of weight concerns (WCS; mean= 47.9 vs mean=40.5; *P*=.03) compared with children of nonparticipating parents. This might be indicative of higher levels of impairment of children of participating parents. In addition, when parent-reported and daughters’ self-reported weight loss was compared, the discrepancy between the 2 estimates was significantly smaller for nonparticipating parents compared with participating parents (1.2 vs 2.15 kg; *P*=.03).

### Intervention Adherence and Acceptance

On average, intervention group parents opened 28% of program pages (median 16%), 2.7 of 6 sessions (median 2.0), and logged on to the program 3.4 times (median 3.0; range 0-11). In total, 29% of randomized parents never logged on to the program at all and only 16% opened more than 75% of program pages. However, participating intervention group parents overall rated the program quite favorably as *good* (mean=2.2; SD=0.94; range=1-5; scale from 1 [*very good*] to 6 [*very poor*]), rated the program content and group moderation on average between *good* and *very good* (means 1.8 and 1.7, respectively), and reported they would *very much* recommend the program to other parents of at-risk children (mean=3.60; SD=0.74; range=2-4; scale scores from 1 [*not at all*] to 4 [*very much*]).

### Primary and Secondary Outcomes

Results of ITT analyses of primary and secondary outcomes based on daughters’ self-report are summarized in Table 2. Of the primary outcomes, only 1 significant difference between the intervention and the control group was found: between preintervention and 12-month FU, girls of the intervention group gained significantly more and faster weight as indicated by change in percentage of EBW compared with girls in the control group. There was a significant time-squared by group interaction indicating the effect of intervention on EBW percentage was curvilinear. The greatest effect of intervention on EBW percentage occurred early during the observation period. The total effect of intervention on EBW percentage can be estimated by adding the estimated group by time interaction effect with the group by time squared interaction effect (21.0–15.5=5.5%), which estimates a 5.5% greater increase in EBW percentage in intervention group participants compared with control group participants. The effect size (*d*=*0*.42) is in the small to medium range. No other significant differences were found between groups on child- and parent-reported secondary outcomes. In both groups, no new onset full-syndrome DSM-IV diagnoses of AN were observed over time.

Multimedia Appendices 1 and 2 provide means and SDs for primary and secondary outcomes for both groups (based on daughters’ self-report and parental report) at all assessment points.

### Parent-Reported Reasons for Unwillingness to Participate

Whenever possible, we asked the parents who declined participation in the study after being told that their daughters had screened positive to give reasons for their unwillingness to participate (Table 3). The majority of these parents responded that they did not perceive the identified risk factors and early symptoms in their daughters as problematic and, accordingly, participation in a preventive intervention as necessary or useful. Other frequently reported reasons were lack of time and daughter’s own unwillingness to participate. A relatively large proportion of parents also reported *all-clear* given by the pediatrician (ie, the pediatrician did not consider the daughter’s weight loss problematic or explicitly advised parents not to participate in the study). In a considerable proportion of cases, parents also reported a change in measures included to define risk status in the screening (eg, a weight gain after screening) or revised the previously reported screening criteria (eg, family history of ED). Some parents, however, also seemed to be afraid to worsen the current condition of their daughter (“let sleeping dogs lie”) by getting engaged in the intervention or reported too many other current problems to get further engaged. A small proportion of children were reported to be already in treatment because of eating or other mental health problems.

**Table 2:** Intervention effects on outcome variables estimated with mixed-effects models.

Effect		Group*time (95% CI)	*t* ratio	*P* value	Cohen *d*
Percentage of expected body weight		21.0 (5.81 to 36.13)	2.76	.007	0.42^a^
Group*time squared effect		−15.5 (−26.6 to −4.49)	−2.81	.007	0.42^a^
	Excessive exercise	0.82 (−3.97 to 5.62)	0.34	.73	0.09
	Weight Concerns Scale	2.02 (−8.03 to 12.08)	0.40	.69	0.08
	EDI-2^b^ bulimia	2.32 (−1.95 to 6.61)	1.09	.28	0.29
	EDI-2 drive for thinness	1.86 (−1.05 to 4.77)	1.28	.21	0.27
	EDI-2 body dissatisfaction	2.77 (−0.36 to 5.89)	1.76	.08	0.34
	EDE^c^ total score	0.04 (−0.41 to 0.5)	0.19	.85	0.04
	EDE dietary destraint	−0.04 (−0.63 to 0.55)	−0.13	.90	−0.03
	EDE eating concern	0.12 (−0.53 to 0.77)	0.38	.71	0.13
	EDE weight concern	0.03 (−0.72 to 0.77)	0.07	.94	0.02
	EDE shape concern	−0.03 (−0.73 to 0.67)	−0.09	.93	−0.02

^a^Estimated Cohen *d* for percentage of expected body weight is the sum of the standardized effects for group by time plus group by time squared.

^b^EDI-2: Eating Disorder Inventory.

^c^EDE: Eating Disorder Examination.

**Table 3:** Parent-reported reasons for declining participation (N=137 parents; multiple answers possible).

Parent-reported reasons for declining participation	Endorsements, n
Do not see risk factors and symptoms as problematic	89
Pediatrician does not see a problem or does not recommend study participation	18
Lack of time	16
Interview canceled, not attended, no response, or no reason given	30
Daughter declines participation	13
Change in risk status since screening (weight gain, exercise, and family history of eating disorder)	15
Too many other problems	5
Afraid to raise awareness for eating disorder problems	5
Currently in treatment for eating disorder or other mental health problem	3

## Discussion

### Principal Findings

The objective of this study was to evaluate the efficacy of a parent-based, targeted preventive intervention for children and adolescents at risk for AN compared with an assessment-only control group. The intervention was specifically developed to target early symptoms and potential risk factors for AN that distinguish E@T from other preventive interventions for ED. It also incorporated elements from the current most promising treatment approach for adolescents with AN, that is, family-based treatment. This trial was preceded by a pilot study with overall encouraging results conducted in both the United States and Germany. We found that—over the course of the study and at the 12-month FU and based on ITT analyses—at-risk girls, whose parents had participated in E@T, gained significantly more and faster weight based on change in percentage of EBW compared with girls in the control group. Although the effect size of this change is in the small to medium range, previous Web-based prevention trials for ED in general usually do not find differences in BMI [17,46,47]. In addition, low weight was one of the risk factors or early symptoms we hoped to change through the intervention. However, these results must be considered in the context that few parents were willing to enroll and engage in the study and no other significant effects on primary or secondary outcomes were found in the ITT analyses. In interpreting the results, it is also important to note that means of outcome measures included in the screening (ie, WCS and EDI drive for thinness) dropped between screening and preintervention assessment. Furthermore, ED and weight-related measures improved in participants of both groups who completed postintervention and FU measures, which limit the potential to see differences. The reasons for the improvement in the control groups are not known but might be indicative of regression to the mean effects. Participants underwent detailed ED interviews after initial screening over the course of more than a year, which in itself may have raised awareness for risk factors and symptoms in parents and may have contributed to improvements in both groups.

### Limitations

The results of this study need to be discussed in the context of the following limitations: (1) small sample size because of low screening completion rates possibly resulting in too little power to establish efficacy; (2) low rates of eligible participants agreeing to participate; (3) low parental engagement in the intervention; and (4) high dropout rates, which again may have affected power, randomization and, thus, also conclusions drawn from the analysis. Parents’ (low) willingness to partake in a Web-based intervention aimed at reducing their daughter’s risk of AN was the kernel of this study and warrants further exploration. Of screens distributed in schools, 35.7% were returned. At the beginning of recruitment, this rate was 20% but was increased by a number of strategies (eg, letter of recommendation of school authorities directed at individual schools, increasing awareness for the study by increased press releases, and offering incentives to girls). Furthermore, even for girls identified as being at risk, parental willingness to participate in the study was low. Only about half of identified families provided contact information, and of those contacted, only about 16% could be randomized. Although parents receiving the intervention, on average, rated the program favorably, they accessed less than a third of all program pages and less than half of the sessions. Using a standardized measure of engagement might have provided information to explain this discrepancy. Adherence is a well-known problem for Web-based interventions in general [48]. However, compared with targeted preventive interventions for ED, in which adherence usually ranges between 50% and 80% [49], adherence to E@T was clearly lower. Along with low engagement rates in the intervention, the study was also characterized by high dropout rates, that is, over 50% in the control group and 65.6% in the intervention group. These rates exceed dropout rates of both targeted intervention trials for ED in general [46,47] and of those reported for family-based treatment trials for AN, which average between 15% and 25% [50-52].

### Comparison With Prior Work

In the absence of specific, parent-based prevention trials for girls at risk for AN, we can only compare our results with more general, parent-based preventive studies. For example, compared with parents referred to outpatient treatment for child conduct problems in the validation sample of the PMI [44], parental motivation in this preventive trial was much lower. As included children had not already developed a mental health problem requiring treatment, parents may have been more reluctant to engage in the intervention. In our pilot study [29], we also found a positive correlation between daughter’s risk status and parental engagement: at the US site, parents of children who already met criteria for AN showed higher levels of engagement with the intervention than those of children at risk for AN. A recent review of interventions involving parents that aim to prevent body dissatisfaction or eating disorders [53] identified 20 studies, 12 of which presented data on the effects of involving parents in prevention programs. A quarter of these studies revealed significant problems with parental recruitment and motivation, despite daughters being screened at-risk [29,54,55]. Although Hart et al [53] concluded that preventive interventions involving parents may have some benefit, they also expressed concern over the finding *that measuring and communicating a child’s at-risk status does not appear to improve parent engagement with prevention programs*.

On the other hand, even with pediatric long-term medical conditions, such as asthma, cystic fibrosis, HIV, diabetes [56], or life-threatening conditions requiring pediatric organ transplantation [57], parents’ and caregivers’ nonadherence to prescribed treatments is reported to be a common problem. Low adherence or denial may therefore represent a more general problem of parents when confronted with chronic or potentially threatening health conditions of their child.

Given the low parental engagement, participation, and completion rates, this sample is likely to be biased toward parents who are more willing to respond to a perceived risk for AN in their daughter (eg, higher motivated parents or parents more willing to acknowledge these risks). Thus, the observed intervention effect on percentage of EBW likely applies to this group of parents. This interpretation is supported in part by the reasons parents gave for unwillingness to participate that we gathered from parents that could be contacted. The majority of these parents did not consider the identified risk factors and early symptoms in their daughters as severe enough to get engaged or do so despite their daughter’s refusal to participate. As included children were, on average, at the time of the preintervention assessment not markedly underweight, parents of these normal-weight children fulfilling the weight loss criterion may have not perceived other risk factors, such as increased weight concerns, as problematic.

The comparison of parents willing to participate and those refusing to participate, on the other hand, shows that daughters of participating parents had even higher levels of ED-related impairment and parents unwilling to participate may underestimate their daughters’ weight loss. Thus, although parents of daughters with higher levels of ED risk factors and symptoms were more willing to participate in the study, symptoms of AN may have needed to be even more pronounced for most parents to engage at all or to engage more consistently in a preventive intervention. Alternatively, given the insidious course AN onset can take, parents may have needed more time and further evidence to realize and accept these risk factors and symptoms to motivate their engagement.

A recent systematic review [58] suggested 6 categories of reasons for parents’ and caregivers’ nonadherence to prescribed treatments in pediatric long-term medical conditions, including concerns or fears of the condition or the recommended treatment, difficulty following the treatment regimen, children’s resistance to treatment, perceived threats and strains to family relationships, parental priorities to preserve *normal life*, and (negative) input from and relationship with health professionals. Some of these reasons may explain parents’ low engagement and adherence in this study. Future studies, therefore, should address these potential barriers to engagement and parental level of readiness to engage more explicitly.

### Conclusions

In conclusion, the intervention showed small effects on only 1 outcome, and given the few parents who were willing to enroll and engage in the study, the intervention does not have the potential for wide-scale acceptance as we hoped for. It may be more beneficial for parents willing to face their daughter’s initial problems, when these have become more pronounced or as the first step for parents of children with full syndrome AN before getting engaged in outpatient or inpatient treatment. However, this will need to be demonstrated in subsequent studies. The parent-based intervention did show some promise for the subsample of children of parents willing to engage in the assessments and in the intervention, even when only administered in a relatively small dose. Together with detailed interviews and feedback on ED risk factors and symptoms, children at risk may benefit from the intervention. Next steps in developing a population-based intervention targeting parents with children at risk for AN would be to (1) consider reasons why parents did not find the identified risk factors compelling, (2) develop better and more effective ways to convey information about risk factors, and (3) identify strategies to resolve parents’ concerns with engagement. Preventive interventions for ED may generally need to educate parents more explicitly about the potential dangers of early signs of disordered eating, such as dieting or weight loss in a child. Finally, a strategy for making the intervention more readily accessible is needed; although we had hoped to offer the original intervention as part of the curriculum in the participating schools, we were ultimately not allowed to do so. By offering the program in a systematic normative manner to parents in the school setting, potential avoidance and stigma, which likely interfered with engagement in the intervention, might be reduced.

## Appendix

Multimedia Appendix 1 Primary and Secondary Outcomes (Daughters` Self-Report).

Multimedia Appendix 2 Secondary Outcomes (Parent-Reported Outcomes).

## Abbreviations

AN: anorexia nervosa
BMI: body mass index
DSM-IV: Diagnostic and Statistical Manual of Mental Disorders-Fourth Edition
E@T: Eltern als Therapeuten
EBW: expected body weight
ED: eating disorder
EDE: Eating Disorder Examination
EDI: Eating Disorder Inventory
FU: follow-up
ITT: intention-to-treat
MPS-F: Frost Multidimensional Perfectionism Scale
PMI: Parent Motivation Inventory
WCS: Weight Concerns Scale
