# Supplementary material for: Efficacy of a Parent-Based, Indicated Prevention for Anorexia Nervosa: Randomized Controlled Trial
Source: J Med Internet Res. 2018 Dec 14;20(12):e296. doi: 10.2196/jmir.9464 (PMC6315221; doi:10.2196/jmir.9464)
Supplement: Multimedia Appendix 1 [file jmir_v20i12e296_app1.pdf]

			Primary Outcomes				
			T0 (Screening)	T1	T2	T3	T4
% EBW	E@T	N	32	32	16	12	11
		mean	94,92	98,77	101,84	103,17	103,40
		SD	12,74	12,30	13,52	16,80	17,81
	CG	N	33	34	26	18	14
		mean	95,12	99,13	96,45	93,66	93,50
		SD	11,69	13,43	12,34	11,56	10,71
Weight concerns (WCS)	E@T	N	32	32	16	12	11
		mean	47,81	39,32	34,69	29,86	26,21
		SD	24,56	22,47	18,36	18,92	19,75
	CG	N	33	34	26	18	15
		mean	53,48	40,24	35,90	26,39	20,33
		SD	26,45	26,94	27,07	23,15	20,89
EDE restraint	E@T	N		32	16	12	11
		mean		1,22	0,86	0,63	0,62
		SD		1,30	1,27	0,93	0,90
	CG	N		34	26	18	15
		mean		1,31	1,27	0,88	0,53
		SD		1,39	1,68	1,32	0,95
Excessive exercise	E@T	N		14	5	4	5
		mean		10,71	24,40	9,50	10,00
		SD		10,34	5,77	13,23	14,14
	CG	N		15	7	8	2
		mean		11,53	11,00	7,13	17,50
		SD		10,72	10,39	6,96	3,54
			Secondary Outcomes				
			T0 (Screening)	T1	T2	T3	T4
EDE weight concern	E@T	N		32	16	12	11
		mean		1,81	1,44	1,52	1,33
		SD		1,54	1,32	1,63	1,24
	CG	N		34	26	18	15
		mean		1,83	1,59	1,30	0,97
		SD		1,59	1,69	1,38	1,33
EDE shape concern	E@T	N		32	16	12	11
		mean		1,86	1,57	1,87	1,39
		SD		1,54	1,35	1,65	1,00
	CG	N		34	26	18	15
		mean		2,03	1,84	1,41	1,11
		SD		1,59	1,80	1,41	1,41
EDE eating concern	E@T	N		32	16	12	11
		mean		0,73	0,49	0,50	0,62
		SD		0,92	0,58	0,94	1,55
	CG	N		34	26	18	15
		mean		0,84	0,80	0,47	0,29
		SD		1,03	1,29	0,69	0,42
EDI-2 drive for thinness	E@T	N	32	32	16	12	11
		mean	23,50	17,16	14,63	15,92	14,00
		SD	9,19	6,19	5,19	5,65	4,10
	CG	N	34	34	26	18	15
		mean	24,21	18,56	17,00	14,17	12,13
		SD	10,06	7,44	8,04	7,52	6,05
EDI-2 body dissatisfaction	E@T	N		31	16	12	11
		mean		19,77	18,31	19,25	16,55
		SD		7,14	8,08	8,34	4,63
	CG	N		34	26	18	15
		mean		21,47	20,38	17,11	14,00
		SD		8,74	9,36	8,84	5,86
EDE total	E@T	N		32	16	12	11
		mean		1,41	1,09	1,13	0,99
		SD		1,20	1,02	1,14	0,96
	CG	N		34	26	18	15
		mean		1,50	1,38	1,02	0,73
		SD		1,32	1,54	1,10	0,91
Body mass index (BMI)	E@T	N	32	32	16	12	11
		mean	18,43	19,32	20,35	20,85	21,21
		SD	2,84	2,69	2,99	3,55	3,92
	CG	N	33	34	26	18	14
		mean	18,37	19,26	19,01	18,70	18,82
		SD	2,39	2,61	2,57	2,36	2,21
