# Supplementary material for: Efficacy of a Parent-Based, Indicated Prevention for Anorexia Nervosa: Randomized Controlled Trial
Source: J Med Internet Res. 2018 Dec 14;20(12):e296. doi: 10.2196/jmir.9464 (PMC6315221; doi:10.2196/jmir.9464)
Supplement: Multimedia Appendix 2 [file jmir_v20i12e296_app2.pdf]

			T1	T2	T3	T4
Parent motivation (PMI)	E@T	N	31			
		mean	79,84			
		SD	23,17			
	CG	N	34			
		SD	21,9			
		mean	76,24			
Child EDE restraint	E@T	N	32	17	12	11
		mean	0,99	0,38	0,73	0,62
		SD	1,41	0,58	1,14	0,86
	CG	N	34	26	18	15
		mean	0,84	0,70	0,66	0,34
		SD	1,39	1,41	1,53	1,09
Child EDE eating concern	E@T	N	32	17	12	11
		mean	0,36	0,18	0,45	0,47
		SD	0,78	0,31	0,68	0,59
	CG	N	34	26	18	15
		mean	0,34	0,31	0,21	0,08
		SD	0,74	0,83	0,38	0,22
Child EDE weight concern	E@T	N	32	17	12	11
		mean	1,48	1,33	1,39	1,64
		SD	1,32	1,10	1,19	1,33
	CG	N	34	26	18	15
		mean	1,38	1,11	0,94	0,93
		SD	1,55	1,32	1,09	1,04
Child EDE shape concern	E@T	N	32	17	12	11
		mean	1,42	1,48	1,75	1,77
		SD	1,17	1,15	1,33	1,29
	CG	N	34	26	18	15
		mean	1,40	1,28	1,03	1,01
		SD	1,52	1,44	1,08	1,15
Child EDE total	E@T	N	32	17	12	11
		mean	1,06	0,84	1,08	1,13
		SD	1,05	0,63	0,89	0,87
	CG	N	34	26	18	15
		mean	0,99	0,85	0,71	0,59
		SD	1,22	1,17	0,94	0,82
Child excessive exercise	E@T	N	7	5	2	4
		mean	10,00	13,40	14,00	7,00
		SD	10,66	12,16	19,80	14,00
	CG	N	8	4	4	5
		mean	4,50	10,00	8,00	5,20
		SD	5,07	14,14	8,08	7,43
